# Effect of Handler Knowledge of the Detection Task on Canine Search Behavior and Performance

**DOI:** 10.3389/fvets.2020.00250

**Published:** 2020-05-27

**Authors:** Mallory T. DeChant, Cameron Ford, Nathaniel J. Hall

**Affiliations:** ^1^Department of Animal and Food Sciences, Texas Tech University, Lubbock, TX, United States; ^2^Ford K9, Las Vegas, NV, United States

**Keywords:** detection dog, handler bias, behavior, olfaction, double-blind

## Abstract

Detection dogs are commonly trained and tested under conditions in which the handler or the evaluator knows the true presence or absence of a target odor. Previous research has demonstrated that when handlers are deceived and led to believe that a target odor is present, more false alerts occur. However, many detection teams operate under *unknown* conditions, and it remains unclear how handler knowledge (or lack thereof) of odor presence/absence influences the dog's behavior. The aim of this study was to evaluate if knowing the number of hides placed influenced detection dog performance in an applied search environment. Professional (*n* = 20) and sport (*n* = 39) detection handler-dog teams were asked to search three separate areas (area 1 had one hide, area 2 had one hide, area 3 was blank). Handlers in the Unknown Group were not told any information on the number of hides whereas the Known Group were told there was a total of two hides in the three areas. The sport Unknown Group spent a longer duration (69.04 s) searching in area 3 compared to the sport Known Group (*p* = 0.004). Further, sport dogs in the Unknown group looked back to the handler more frequently. When a miss did occur, dogs of both sport and professional handlers showed an increase interest in the location of the target odor compared to a comparison location. Critically, however, there was no difference in false alerts between the Known Group and Unknown Group for sport or professional handlers. In a second experiment, fourteen professional, and thirty-nine sport teams from Experiment 1 conducted an additional search double-blind and an additional search single-blind. Both sport and professional-handler dog teams had statistically similar accuracy rate under single and double blind conditions. Overall, when handlers knew the number of hides, it led to significant changes in search behavior of the detection team but did not influence the overall false alert rates.

## Introduction

Dogs have been utilized for a myriad of professional detection jobs for items such as explosive devices (1–3), narcotics (4, 5), semen stains for crime scenes (6), human odor (7–11), cancer (12–14), and conservation (15–19). Dogs are also utilized for non-professional detection jobs such as bird hunting (20) and sport detection in the United States and in growing sport detection dog organizations across the world. Sport detection dogs typically detect essential oils and compete in sanctioned events through the American Kennel Club, The National Association of Canine Scent Work, and United Kennel Club. Whether the dog is utilized for professional or non-professional detection work, a handler always works with the dog.

The focus of detection work is typically on the detection dog itself; however, an undeniable bond between the handler and the dog could influence performance (21). Handlers have the responsibility to recognize and call the dog's change in behavior toward an odor, or trained alert, to locate the target source. Failure to call an alert could result in a missed target source which can have detrimental implications for certain professional (e.g., explosive or live find search and rescue) detection dogs. Further, calling an alert when a dog is not showing the appropriate alert behavior or unintentionally cuing a dog to alert could lead to unnecessary emergency (e.g., explosive dog) or improper search (e.g., narcotics dog).

Maintaining a strong trained alert behavior in an operational setting is critical because detection dogs are often subjected to stressful situations in which they work (15). To prevent deterioration of the alert behavior and to maintain olfactory performance, handler-dog teams train on a regular basis, frequently referred to as “maintenance training” (22). Moser and McCulloch (23) reviewed various training details from publications in which dogs were trained to detect cancer. Training regimens range from a frequency of 15–30 times per day (24, 25) to a duration of 1–2 hours per day (26). The ranges used by typical professional detection dogs have not yet been clearly reported but are likely variable depending on the type of work. Non-professionals such as sport detection handler-dog teams commonly train once a week but may vary depending on opportunities available in their city. Frequent training sessions could potentially maintain a strong alert behavior and increase accuracy in odor discrimination; however, there is limited research on how different maintenance training regimens influence detection performance.

Typically, a dog remains with the same handler, or handlers, throughout their working life; however, there are some circumstances (e.g., retirement or death) where a new handler could take possession of the dog. For teams that have a consistent one-handler to one-dog working relationship, maintaining the same handler-dog team is preferred because changing handlers impacts performance by increasing response time (27), the dog becoming distracted more often, and potentially less accurate (28).

Handlers, however, may potentially negatively impact working performance by unintentionally cuing the dog (29–32). A classic example of unintentional postural and facial cues is the famous “Clever Hans” example, in which a horse's incredible skills was later demonstrated to be remarkably controlled by unintentional cues (33). A similar phenomenon could occur with handler-dog teams where the dog responds to unintentional handler cues (34). Dogs, in particular, are quite adept at reading human communicative cues (35, 36), and perhaps may learn to utilize these cues during a search, even when unintentional by the handler.

In a critical study, Lit et al. (31) found that police canine handler belief that an odor source was present led to significant rates of false alerts by the dogs. In this study, handlers were deceived by informing them that a target odor was placed in a certain location, when marked. Importantly, no target was actually placed, but instead, sometimes a non-target distractor odor was presented (31). This was contrasted to areas in which no obvious maker was placed for the handler. Overall, more false alerts occurred when the handler was led to believe a target odor was present (31).

Importantly, no follow-up to this study has been conducted. There remain several limitations that require further study. First, Lit et al. (31) deceived handlers as to the presence of an odor which caused handlers to influence the dog's indication; however, a double blind or unknown condition is perhaps more realistic of an operational setting, when handlers are unsure of target odor presence, rather than being told by a researcher that target odors are present. Second, although more false alerts were called (i.e., the handler is the person “calling” the alerts), there was no direct behavioral observation of the dog to investigate the dog's behavior and to what degree they showed a true alert (i.e., the dog displayed the behavior trained for indicating a target odor at a specific location such that the handler can recognize).

The objectives of this study were to determine whether knowing the number of odor sources prior to a real-life scenario search influenced detection dog outcomes and to evaluate if single-blind or double-blind searches influenced detection dog outcomes. In addition to professional handlers, sport handlers were also utilized to increase the sample size of detection dog handlers. Dog behavior during each study was coded by video, and certain team descriptor covariates (i.e., handler training experience, frequency of double-blind training, and frequency of blank training) were utilized as covariates for data analysis.

## Materials and Methods

This study was conducted at various field locations and was approved as an observational Institutional Animal Care and Use protocol. The Institutional Review Board at Texas Tech University approved this study (IRB2019-501). All participants provided written consent. Recruitment statements were sent out via email to various professional detection dog agencies and sport scent detection trainers following an approved script. Participants were volunteers and completed a survey following the search. There were two components to this study: Experiment 1) a three area search with varying levels of knowledge of the number of target odors present and Experiment 2) a single-blind and double-blind search. This study was conducted in six cities across the United States and at seven different facilities that permitted dog search teams during after-hours. Specific cities and facilities will remain confidential to maintain anonymity of professional and sport handler-dog teams. Search areas were chosen and secured for training by the local organizations (and was therefore not experimentally controlled). Size of the search areas were kept as consistent as possible across locations (e.g., typical search was in a standard classroom approximately 85 m^2^). Twenty professional and 39 sport handler-dog teams were recruited for Experiment 1. Fourteen professional and 39 sport handler-dog teams from Experiment 1 participated in Experiment 2. All searches were video recorded for data collection.

### Handler-Dog Teams

The handler-dog teams that participated were divided into professional and sport handler groups, based on self-report. All searches were typical of an operational search or a scent detection trial. All sport detection searches utilized birch essential oil. Professional detection searches utilized smokeless black powder, ammonium nitrate, trinitrotoluene dynamite, composition-4, cast booster, detonation cord, marijuana, methamphetamine, cocaine, heroin, bed bugs, kerosene, and 75% evaporated gasoline depending on the type of detection dog and the agencies' training aids provided. This variability in target odor was required given the different agencies and odor stimuli to which they had previously trained the dogs. Training aids were supplied by the respective departments. All distractors utilized in searches were cotton balls and plastic gloves. Dogs were allowed on or off-leash depending on handler preference. Each search was timed for duration but did not have a time limit.

### Experiment 1: Knowledge of the Number of Hides Present

Three indoor and temperature regulated areas were utilized for three searches. The facility used was secured and organized by the professional or sport team organization, and therefore little control over specific facilities was available, but most were schools or business with similar sized rooms (~83 m^2^). To control within a given study-site, the three areas were selected based on identifying three approximately similar sized rooms or parts of a room, as facility geometry allowed. The professional handler-dog teams searched the entire area for the target odor provided by the organization's trainer that was concealed in a small tin not visible to the handler or canine and was ~50 cm from the ground. The sport handler-dog teams searched containers provided by the trainer (small cardboard boxes and plastic shoe bins) placed on the floor spread across a similar area. The target odor was also provided by the trainer and was concealed in a small tin not visible to the handler or canine. Twenty professional handler-dog teams and 39 sport handler-dog teams were recruited. Handlers were instructed to search and clear each area before proceeding to the next area. Handlers therefore determined when to move from one area to the next. A handler was instructed to call an alert by placing their hand up and saying “alert.” The experimenter would then provide feedback as to whether it was correct or a false alert. No feedback was given if a dog missed a target, until after the study completion. The primary experimenter video recorded the dog through the search from as far back as possible and was knowledgeable of the target odor placement. Handlers in the Unknown Group (Professional *n* = 10; Sport *n* = 19) were instructed from Script 1 (see below) and Known Group (Professional *n* =10; Sport *n* =20) were instructed from Script 2 (see below). Areas were set up as follows: area 1 contained one target odor, area 2 contained one target odor, area 3 had no target odor and plastic gloves as a distracting odor. Detailed data recorded during the searches are defined in Table 1 (see below).

**Table 1:** Data recorded during three area searches.

**Term**	**Definition**	**Area recorded**	**ICC**
Total search duration	Start of search to when handler called “clear”	Area 1, 2, 3	0.99
Hit	Handler called alert when dog is at target odor source	Area 1, 2	NA
False	Handler called alert when dog is not at target odor source	Area 1, 2, 3	NA
Correct rejection	Handler correctly did not call an alert	Area 3	NA
Miss	Handler did not call an alert and dog did not locate target odor	Area 1, 2	NA
Target investigate duration	If dog “misses,” duration of sniffing time at target odor source	Area 1, 2	0.83
Non-target investigate duration	If dog “miss,” duration of sniffing time at selected non-target area along search path	Area 1, 2	0.92
False alert duration	Start of search to when handler called alert and dog is not at target odor source	Area 1, 2	0.92
Hit duration	Start of search to when handler called alert and dog is at target odor source	Area 1, 2	0.98
Lookback	Number of times the dog turned their head back to look at the handler	Area 1, 2, 3	0.97

**Script 1**. Unknown Group instructions for three area searches.

*You will be searching a total of three areas*.

*When your dog alerts, call out the location and I will immediately tell you if it is correct or not*.

**Script 2**. Known Group instructions for three area searches.

*You will be searching a total of three areas*.

*There are exactly 2 target odors in total, over the three areas*.

*One room has no target odor in it*.

*When your dog alerts, call out the location and I will immediately tell you if it is correct or not*.

### Experiment 2: Single-Blind and Double-Blind Comparison

Two indoor and temperature regulated areas were utilized for single-blind and double-blind searches. Fourteen professional handler-dog teams and 39 sport handler-dog teams were recruited from Experiment 1 immediately after (same day). All handlers were instructed from Script 3 (see below). Areas were set up as follows: single-blind contained one target odor and a cotton ball as a distractor odor, double-blind contained one target odor and a cotton ball as a distracting odor. Detailed data recorded during the single-blind and double-blind searches are defined in Table 2 (see below). Accuracy was defined if the handler-dog team correctly called an alert to the target odor. Trials were videotaped via a tripod positioned to record the entire search area. All other experimental arrangements such as the facilities used were identical to Experiment 1.

**Table 2:** Data recorded during both single-blind and double-blind searches.

**Term**	**Definition**
Hit	Handler called alert when dog is at target odor source
False	Handler called alert when dog is not at target odor source

**Script 3**. Handler instructions for single-blind and double-blind searches.

*Mallory will be the judge and can help you through this experiment*.

*Mallory will know where the odor is located*.

*For the single-blind search, Mallory will be watching the search*.

*For the double-blind search, Mallory will be facing a wall and will not be watching the search*.

*When your dog alerts, call out the location and Mallory will immediately tell you if it is correct or not*.

### Survey

A custom survey was created using Qualtrics but distributed via paper copy (www.qualtrics.com; see Supplementary Material for complete survey). Dog handlers (de-identified via a participant ID number) answered questions about their dog, whether they were a professional or sport handler (do they receive money for detection services or not), detection training frequency (reported as 1: daily, 2: 4–6 times a week, 3: 2–3 times a week, 4: once a week, 5: 2–3 times a month, 6: once a month, 7: less than once a month), years of experience, listed the odors the dog is trained on, frequency of double-blind training (reported as 1: always, 2: most of the time, 3: about half the time, 4: sometimes, 5: never), frequency of conducting blank searches (reported as 1: multiple times a training session, 2: once a training session, 3: every other training session, 4: every 3–5 training session, 5: almost never, 6: never), the dog's alert behavior, handler belief that target odor was present in area 3 (reported as 1: strongly agree, 2: agree, 3: somewhat agree, 4: neither agree nor disagree, 5: somewhat disagree, 6: disagree, 7: strongly disagree), and survey measures of canine behavior which will be analyzed later with a larger sample. Handlers were given multiple choice options (they could select more than 1) and an optional fill in the blank. The survey was administered to the handlers after completion of study 1 and study 2 via a paper copy. Handlers were not required to answer all of the questions. An ID was given to the survey to correspond to the video from study 1 and study 2.

### Hypotheses

Based on previous research indicating important effects of handlers on dog search performance (27, 28) and that handler belief may factor into this (31) we hypothesized that individuals' knowledge regarding the number of odor hides present, or whether the moderator of the search knew the presence of the hides, would lead to differences in canine performance. We developed the below specific hypotheses in which we expected handler knowledge to influence performance and additional hypotheses related to the dog's search behavior:

Experiment 1

1. Sport and Professional handlers in the Unknown condition would report (via survey) a higher expectation to find an odor in area 3 than the Known group that were previously informed there was a blank room and only a total of 2 hides.
2. Sport and Professional handlers in the Unknown condition will search longer in the final area compared to handlers that Know there is a blank room.
3. When a dog's misses a target odor (in search areas 1 and 2), they would have shown more investigate behavior toward the target odor than a presumably equivalent comparison blank area along the search path.
4. Dogs will make a false alert later in a search compared to a hit in search areas 1 and 2.
5. Dog will look back toward the handler more frequently in search area 3 for handlers that do not know the number of hides.
6. There will be more false alerts when handlers do not know there is a blank room, compared to handlers that Know one room is clear.
7. Training practices, such as frequency of training under double blind conditions or frequency of training with blank searches will influence the effect of the above hypotheses.

Experiment 2

8. Accuracy will be higher in the single blind condition compared to double-blind condition indicating an effect of the presence of a judge or moderator of the search that is knowledgeable of target odor positioning.

### Statistical Analysis

Data from the sport and professional handler teams were analyzed separately because the level of training is different between the teams, the target odors are vastly different in terms of volatility, and the motivation for working with the dog is different. We did, however, compare trends observed across both groups, but did not formally compare these distinct groups. Logistic regression was utilized to compare accuracy in the single-blind vs. double-blind conditions (Experiment 2). Linear mixed model was utilized for all other comparisons (Experiment 1). Analyses were conducted using R [R version 3.5.1, www.r.project.org; (37)] and the lme4 (38) and lmerTest (39) packages. *P*-values and *Z*-tests of the logistic regression model were obtained from the summary function of the lmerTest package. The following were covariates utilized from the survey: years of handler experience with detection dog, frequency of no target odor (blank) training runs, and frequency of double-blind training. Interobserver agreement was calculated for video-scored behavior for 10% of the total number of participants by a coder naïve to the hypotheses. All Interclass correlation coefficient for video coded variables are in Table 1 and were obtained from the ICC function of the psych package using R (40).

To address hypothesis 3 that when a dog misses, they searched the target area longer than a comparison area, the duration of sniffing was recorded by video toward the target odor and a comparison area. The comparison area was chosen by selecting a location that could have equally of held the target odor (e.g., another container or comparable item where a target maybe hidden) was along the same search path (i.e., along the same wall or edge) and was 3 m from the target odor (to avoid cross-contamination). Sniffing duration to both locations was then scored by video.

## Results

### Overall Performance

Table 3 shows an overview of overall performance for sport and professional dogs in each area. Table 3 shows the percentage of handler teams that made each response (out of 20 professional teams and 39 sport teams). Hits and misses were coded as mutually exclusive given only one target odor was present per search area, but false alerts could occur in addition to a hit or miss. Total search duration was coded from the start of the search until the handler cleared the area. Overall, a majority of professional and sport handlers correctly identified the target odor and most searches were completed within 2 min. There were no clear systematic differences in performance across the search areas, and a two sample proportion test indicates that there was no overall statistical difference in the proportion of handlers false alerting between area 2 and area 3 (professional: χ^2^ = 2.00, df = 1, *p* = 0.16; sport: χ^2^ = 2.60, df = 1, *p* = 0.11) or area 1 and 3 (professional: χ^2^ = 2.00, df = 1, *p* = 0.16; sport: χ^2^ = 0.83, df = 1, *p* = 0.36).

**Table 3:** Overview of Professional and Sport dog-handler team performance in each area. Each cell shows the percentage of handlers that made the respective response (out of 20 professionals and 39 sport handlers).

	**Area 1**		**Area 2**		**Area 3**	
	**Professional**	**Sport**	**Professional**	**Sport**	**Professional**	**Sport**
Hits	50%	79.48%	65%	51.28%	N/A	N/A
False alerts	15%	38.46%	15%	30.76%	40%	51.28%
Misses	50%	20.51%	35%	48.71%	N/A	N/A
Correct Rejections	N/A	N/A	N/A	N/A	60%	48.71%
Average search duration (mean (s) ± sd)	95.29 ± 49.48	59.51 ± 40.47	120.54 ± 134.421	67.27 ± 71.55	127.35 ± 79.60	96.04 ± 69.76

### Experiment 1: Knowledge of the Number of Hides Present

#### Hypothesis 1

We tested whether the handler's expectation for an odor being present in the final search area was higher for individuals in the Unknown group, given that prior to area 3, each room contained a target odor, and they had no knowledge of the number of hides compared to the Known group who were informed there was a blank room and only 2 hides. Overall, there was no difference in self-reported handler expectation for odor presence in area 3 (*t* = −0.224, *p* = 0.82).

#### Hypothesis 2

**Figure 1:**
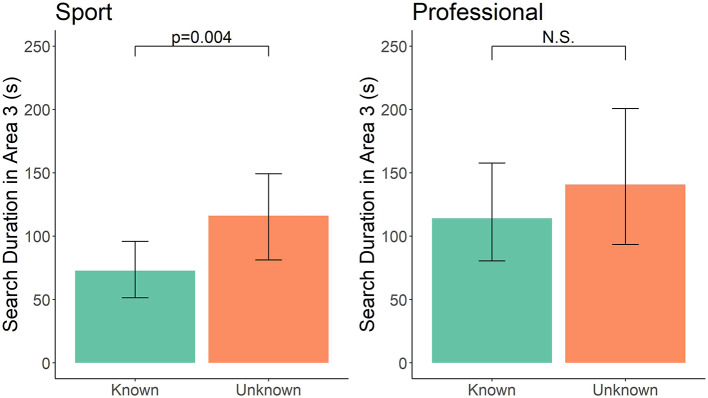
Known vs. Unknown group in sport and professional handler dog teams for total search duration in area 3. Error bars represent 95% confidence intervals.

Although handler expectations did not seem to change, we next evaluated whether handler and canine search behavior changed based on knowledge of the test parameters. Sport-handler dog teams in the Unknown group searched for 69.04 s longer than the known group (*t* = 3.056, df = 33, *p* = 0.004; Figure 1), indicating knowledge that one room was blank reduced overall search time in the final area. In addition, search duration of sport handlers was associated with length of previous training, such that as length of previous experience increased, the dogs searched for a shorter period of time (*t* = −2.268, df = 33, *p* = 0.048). There was no effect of frequency of double-blind training (*t* = 1.198, df = 33, *p* = 0.23) or frequency of blank training runs (*t* = 1.232, df = 33, *p* = 0.22) detected. This analysis, however, includes all participants, whether the individuals accurately found the hides in the first two rooms or not. To evaluate whether this result was the same for participants that accurately identified the first two hides (thus the Known group should be fully knowledgeable that the final room is blank), we subset our data to only these participants. This left eight participants in the Known group and 10 in the Unknown group. In this subset, the trend remained similar, such that handlers in the Unknown group spent ~91 s longer in search area three, although statistically this effect only reached the trend level (*t* = 1.89, df = 16, *p* = 0.08). Professional-handler dog teams in the Unknown group searched for 7.47 s longer than the Known group (similar direction of effect); however, this difference did not reach statistical significance (*F* = 0.035, *t* = 0.189, df = 14, *p* = 0.85; Figure 1). In addition, there was no effect of frequency of double-blind training (*t* = 0.861, df = 14, *p* = 0.40), frequency of blank training runs (*t* = 0.466, df = 14, *p* = 0.64), or years of experience (*t* = 1.65, df = 14, *p* = 0.12). The sample size for professional handlers that correctly identified both finds in the first two areas, however, was too limited to evaluate as a subset as was done for sport handlers.

#### Hypothesis 3

**Figure 2:**
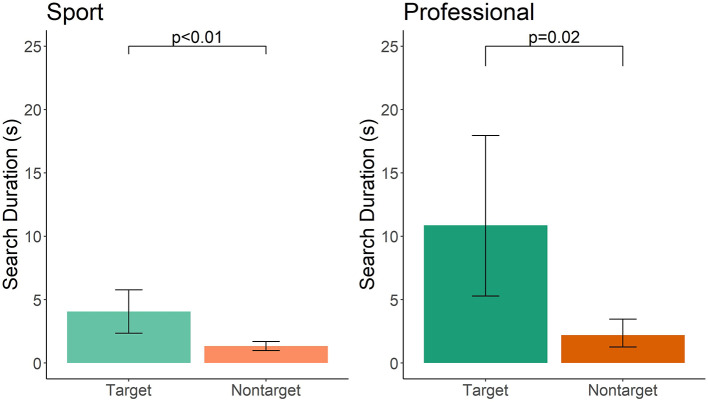
Target vs. non-target investigation duration in sport and professional handler dog teams. Error bars represent 95% confidence intervals.

A miss was scored when the handler did not call an alert when a target odor was present in the search area. To quantify whether the dog showed significant interest to the target odor, but simply did not show a readable alert by the handler, we compared investigation time to the target odor area compared to an equally sized comparison non-target area along the search path. When a miss occurred, sport-handler dog teams investigated the target odor for 3.04 s longer than a comparison non-target location (*t* = −3.11, df = 25, *p* < 0.01; Figure 2). This was not influenced by the frequency of double-blind training (*t* = −0.49, df = 7, *p* = 0.63), frequency of blank training runs (*t* = 0.32, df = 17, *p* = 0.75), or years of experience (*t* = 1.14, df = 13, *p* = 0.27). Professional-handler dog teams investigated the target odor for 9.52 s longer than a comparison non-target location (*t* = −2.52, df = 14, *p* = 0.02; Figure 2). This was not related to reported frequency of double-blind training (*t* = 0.339, df = 8, *p* = 0.74), frequency of blank training runs (*t* = −0.648, df = 6, *p* = 0.54), or years of experience (*t* = 0.85, df = 4, *p* = 0.44). Both sport and professional-handler dogs spent a longer time investigating the target odor than a comparable area along the search path.

#### Hypothesis 4

**Figure 3:**
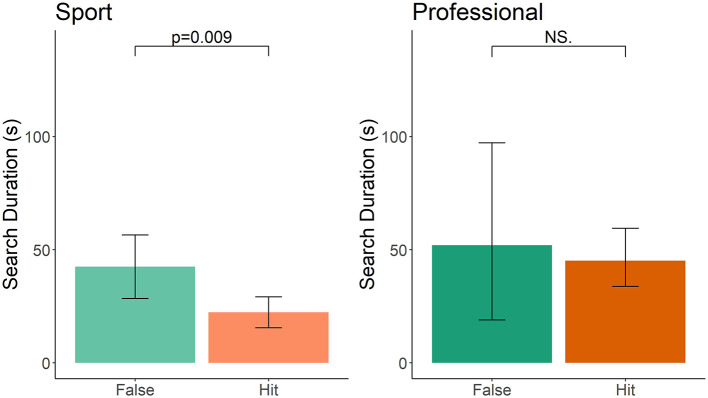
Time of search until either a hit or false alert is called in sport and professional handler dog teams. Error bars represent 95% confidence intervals.

To evaluate if false alerts occurred later in the search than hits, we scored the time from the start of each search in areas 1 and 2 until the first outcome of either a false alert or a hit. We then compared the duration of the search between hits and false alerts. Sport-handler dog teams that false alerted, had on average, searched for 19.70 s longer than when the average hit was called (*t* = −2.682, df = 55, *p* = 0.009; Figure 3). This effect was not associated with the frequency of double-blind training (*t* = 0.201, df = 32, *p* = 0.84), frequency of blank training runs (*t* = 1.617, df = 35, *p* = 0.11), or years of experience (*t* = −0.397, df = 37, *p* = 0.69). Professional-handler dog teams showed no difference between false alert and hit duration (Figure 3; *t* = 0.572, df = 9, *p* = 0.58), which may be due to the fact that only four false alerts were the first thing called in areas 1 and 2 for professionals. No covariates were analyzed for professional-handler teams because of small sample size for comparison.

#### Hypothesis 5

**Figure 4:**
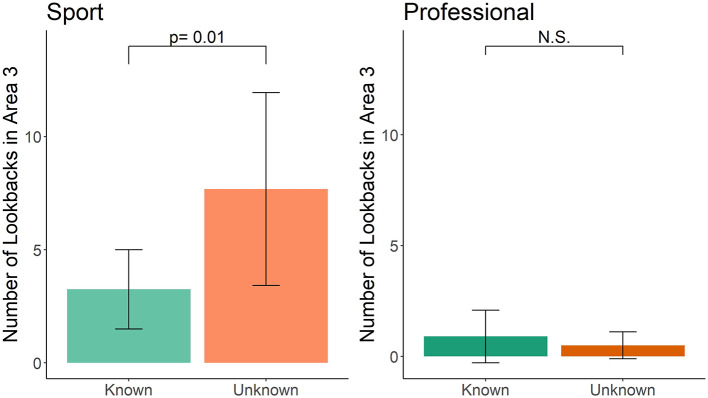
Known vs. Unknown group in sport and professional handler dog teams for number of lookbacks in area 3. Error bars represent 95% confidence intervals.

Sport-handler dog teams in the Unknown group had about three times more lookbacks than the Known group in search area 3 (*t* = 2.522, df = 33, *p* = 0.01; Figure 4). This was not influenced by the frequency of double-blind training (*t* = 1.173, df = 33, *p* = 0.25), frequency of blank training runs (*t* = −0.515, df = 33, *p* = 0.64), or years of experience (*t* = −1.536, df = 33, *p* = 0.13). Professional-dog teams did not have a difference between the number of lookbacks in the Unknown and Known group (*t* = 0.507, df = 18, *p* = 0.50; Figure 4). No covariates were analyzed for professional-handler teams because of small sample size for the number of dogs engaging in lookbacks (only six professional dogs looked back to the handler in area 3).

#### Hypothesis 6

**Figure 5:**
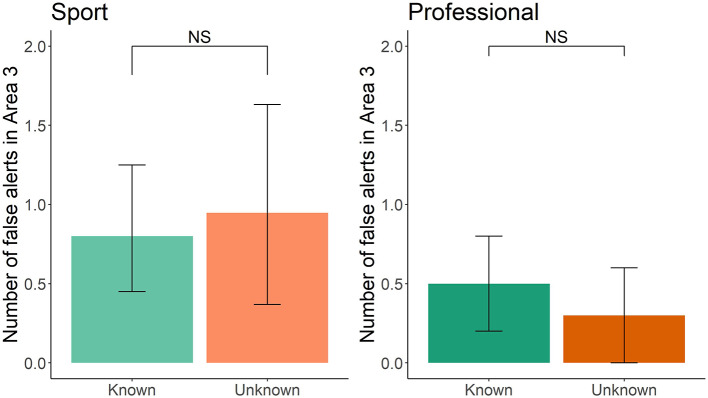
Known vs. Unknown group in sport and professional handler dog teams for number of false alerts in area 3. Error bars represent 95% confidence intervals.

Sport and professional-handler dog teams in the Unknown group did not false alert more in area 3 compared to the Known group (sport: *z* = 0.484, df = 37, *p* = 0.62; professional: *z* = −0.711, df = 18, *p* = 0.47; Figure 5). This was not associated with the frequency of double-blind training (sport: *z* = 0.384, df = 37, *p* = 0.70; professional: *z* = 1.180, df = 18, *p* = 0.23), frequency of blank training runs (*z* = −1.640, df = 37, *p* = 0.10; professional: *z* = −0.434, df = 18, *p* = 0.66), or years of experience (sport: *z* = −0.454, df = 37, *p* = 0.65; professional: *z* = 0.705, df = 18, *p* = 0.48). When restricting analysis to only sport-handler participants that correctly identified the first two targets, there remained no difference in the number of false alerts (*z* = 0.979, df = 17, *p* = 0.33). Interestingly, of the eight sport handlers that accurately identified the two targets in the first two room and knew there were only two targets total and that one area was blank, two of these eight handlers still called false alerts.

#### Hypothesis 8

**Figure 6:**
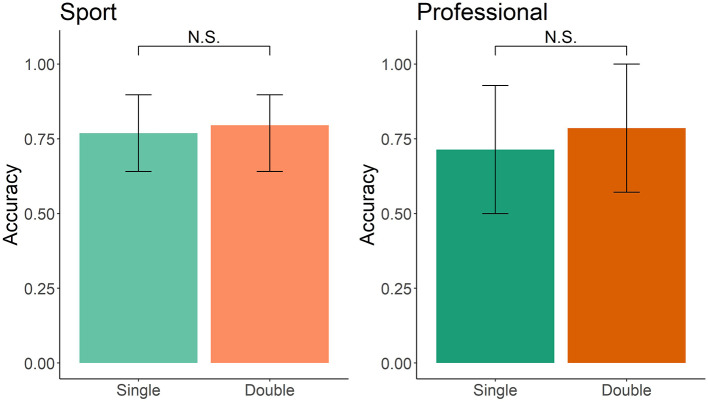
Single-blind vs. double-blind search accuracy in sport and professional handler dog teams. Error bars represent 95% confidence intervals.

In the double blind and single blind search, both sport and professional-handler dog teams had a statistically similar accuracy rate. Logistic regression relating accuracy in the search (1 or 0) to the condition (single blind vs. double blind), showed no significant effect (sport: *z* = 0.295, *p* = 0.76, professional: *z* = 0.435, *p* = 0.43; Figure 6).

## Discussion

Overall, the present results highlight that handler knowledge of the testing parameters influences search behavior, by increasing the search duration in a blank area and increasing the number of lookbacks to the handler by the sport dog. Importantly, however, this did not directly translate to increased rates of false alerts when the number of target odors was Unknown compared to Known. This highlights the need to consider handler knowledge in a search task, as it could lead to a handler limiting search time if the handler believes there is no odor present or extending a search because they believe something is present.

In addition, analysis of canine behavior revealed some interesting findings. First, when dogs did miss a target odor, both sport and professional dogs tended to investigate the location of the target odor more than a comparable area along the search path (from about 3.04 s for sport and 9.52 s for professional). This suggest the dogs did at least somewhat identify the target odor presence through a change in investigation behavior but did not quite show sufficient behavior for an alert. Under controlled conditions, canine investigation behavior does seem to be indicative of whether the response may be correct. Concha et al. (41) found that dogs showed reduced sniffing before a true negative response than before any other response (e.g., false positive, false negative and true positives). Perhaps, together, these results indicate that misses could be limited further by careful observation of this investigation behavior by the handler. Further, increased investigation behavior could be used to indicate to a handler to manipulate the local environment to increase odor availability to the dog (e.g., perhaps by opening a drawer if it is not an explosives dog). It should be noted though, that we did not induce a strong bias in the handler to believe an odor was present. Perhaps, had we done so, any increased investigation time may have led to false alerts, as demonstrated by Lit et al. (31). Thus, interpretation of investigation behavior should be made cautiously, but our present results highlight that during a miss, the target odor was investigated longer than a non-target area.

In addition, analysis of the dog behavior indicated that for sport dogs only, false alerts tended to occur on average later in the search than did hits. This suggests that perhaps if a dog fails to find a target odor after a typical period of time, a false alert may become more likely. This may be an attempt to receive a reinforcer in scenarios in which the dog does not find a target odor, but further testing is required.

In addition, Sport dogs (but not the professional group) in the Unknown group looked back at the handler three times more compared to the Known group in area 3. Previous research has investigated dogs' propensity to look back at an owner when given an “unsolvable” task such as food trapped in a box that can't be opened (42–48). The underlying reasoning dogs engage in high rates of looking back to a human in these unsolvable conditions is still under discussion (44, 47, 48). Interestingly, however, the rate of looking back seems to be inversely related to persistence on the task (46), and positively related to a strong history of training and experience with humans (43). Water search dogs showed higher frequency of looking back compared to pets (42) and another study found that search and rescue, agility and pet dogs show differing patterns of looking back (45). Our blank area 3 for the Unknown group maybe a similar condition to the “unsolvable” task where a dog cannot find a target odor in the search, but the handler is still waiting for the dog to search. In contrast, handlers in the Known group may have been more apt to interpret a lack of alert suggesting that no target odor was present leading to fewer overall lookbacks. Interestingly, we did not see this effect in professional dogs, as few professional dogs engaged in many look backs. Given that the type and frequency of lookbacks to an owner or handler is related to ontogenetic experiences (43, 44, 46), training style differences between sport and professional handlers maybe related to the behavioral differences. However, this was not directly tested, but the present results suggest using blank area searches may be an interesting paradigm to explore dog-handler communication.

Lastly, we did not observe any differences between single-blind and double-blind testing. This suggests that an impartial judge or moderator of the trial may be present without directly influencing performance. Importantly, however, it is critical to note that the judge in the present experiment was a trained researcher familiar with phenomena associated with unintentional cuing. This was done to evaluate whether single-blind testing could be implemented impartially, which is important given that Pfungst (33) himself had trouble limiting unintentional cues given to Clever Hans. Under these conditions, we did not see a bias from the experimenter, but nonetheless, a less impartial judge, or a judge with strong motivations for the canine's performance may still unintentionally provide cues. Thus, it remains critical the impartiality of the judge of a detection dog trial remain under scrutiny and evaluation, but it nonetheless remains possible for an impartial judge to not provide cues to the team.

Across all analyses, we did not formally compare the performance of sport and professional teams given their significantly different backgrounds and variation in target odor volatility. Interestingly, although many comparisons did not quite reach the level of significance in our professional handlers, perhaps due to a smaller sample size, the direction of the effects all remained similar to the sport dogs. This suggests that perhaps sport canine teams may be a good model, where larger samples sizes can be reached quickly, to support research for professional dog teams.

Interestingly, we saw little effect of the years of experience training, reported frequency of double-blind training, or use of blanks in training on overall performance. All together, we only observed years of experience to reduce the duration of search in the blank area, with no other associations reaching the statistical criterion. To our knowledgeable, this was the first evaluation of how these different training methods (i.e., double-blind searchers or blank searches) influence a variety of performance measures. These results, however, may be limited due to sample size, handlers miss remembering reported training practices, or a reporting bias for procedures considered to be optimal. This suggests more rigorous and prospective experimental tests of the effect of these training methods should be conducted to evaluate their effects on operational performance.

There are several important limitations to the present study. First, we did not confirm an increased expectation of a target odor for the Known compared to the Unknown group (Hypothesis 1), although their actual search behavior did reflect this. There are several potential reasons for this finding. First, handlers may have simply mis-remembered their expectation when filling in the survey after the fact. Second, handlers may not have been aware of their changes in expectation for a target odor due to distraction during the search, or perhaps participants anticipated the Experimenters may have been trying to deceive them. Third, perhaps they simply wanted to report that their expectations were not influenced by the knowledge of the search task. Given that we did see behavioral changes between groups suggests that this lack of finding was not critical to the overall results but does suggest that future studies may need to do a better job clarifying the task parameters to participants in a known condition.

Another important limitation is that dogs did miss the target odor in the first two search areas. This likely would influence expectation of handlers in the Known group for search area 3 and introduced noise to the experiment. Nonetheless, we did still see changes in search behavior in area 3, and when we limited our relevant analyses to only the participants that correctly found the target odors in the first two areas, the direction and trend of our results remained similar. This suggests this was unlikely to be a critical limitation. This does suggests, however, that future studies could provide more explicit direction to handlers (e.g., “one odor is present in this area, one is present in this area, and nothing is present here”) and see how that influences the results. For the present study, we opted not to do this as we thought this may be too explicit and would make handlers suspicious of the task. Further, such explicit knowledge rarely, if ever, occurs in the field. However, given that our script (i.e., script 2), did not generate the change in handler expectancy we expected (hypothesis 1), this would be a useful follow-up experiment.

Another important limitation was the relatively smaller sample size of professional handlers compared to sport handlers. This limited the power of some analyses and covariate analysis, but the direction of effects remained congruent with the sport groups. Future studies with increased power with professional groups and sport groups would be important to extend and replicate the present results.

In conclusion, the present results indicate that knowledge of the number of target odors present did lead to changes in behavior of the search team in a blank area. Teams searched the blank area longer, with the dog engaging in more lookbacks to the handler, when they did not know the number of target odors compared to when they did. Overall, however, we did not see handler knowledge about the presence of a blank area (no odor present) to change false alert rates compared to handlers that knew about the blank area. Lastly, we did not see any differences in performance in a single-blind and a double-blind search when an independent experimenter served the role as the trial moderator. Together, these results suggest that handler knowledge of test parameters influences team search behavior but did not lead to changes in false alert rates in a similar manner to previous work. More research is required, however, with varying levels of explicit handler knowledge on search parameters to evaluate its effect on the behavior of the team. Finally, we suggest that sport canine teams may be a good experimental model to evaluate these effects for professional handler teams.

## Data Availability Statement

All datasets generated for this study are included in the article/Supplementary Material.

## Ethics Statement

The studies involving human participants were reviewed and approved by Institutional Review Board at TTU approved this study (IRB2019-501). The patients/participants provided their written informed consent to participate in this study. Ethical review and approval was not required for the animal study because Institutional animal care and use committee reviewed the protocol and it was determined to be an observational protocol. Written informed consent was obtained from the owners for the participation of their animals in this study.

## Author Contributions

MD and NH contributed to the experiment development and analysis. CF contributed to recruiting participants and conducting the experiment. All authors were involved in writing the manuscript.

## Conflict of Interest

The authors declare that the research was conducted in the absence of any commercial or financial relationships that could be construed as a potential conflict of interest.
