# Supplementary material for: Effect of Handler Knowledge of the Detection Task on Canine Search Behavior and Performance
Source: Front Vet Sci. 2020 May 27;7:250. doi: 10.3389/fvets.2020.00250 (PMC7266931; doi:10.3389/fvets.2020.00250)
Supplement: Supplementary file 1 [file Table_1.DOCX]

Area

D: double blind search

S: single blind search

1: area 1 search

2: area 2 search

3: area 3 search

ID

A: Known group

B: Unknown group

All duration time is in seconds (false, hit, investigate, nontarget

“Years Experience” is the number of years the handler has worked with their dog

“Lookback: number of times the dog looked back at the handler during the three area search

“Blank” is the frequency of training trials with no target odors present with the following scale:

1: multiple times a training session

2: once a training session

3: every other training session

4: every 3-5 training session

5: almost never

6: never

“Double” is the frequency of training trials that a judge or trainer (that knows where the odor is hidden) is within visual contact with the handler and dog during trials with the following scale:

1: always

2: most of the time

3: about half the time

4: sometimes

5: never

“Handler belief” is during the search at area 3, did the handler believe there was going to be a target odor hidden with the following scale:

1: strongly agree

2: agree

3: somewhat agree

4: neither agree nor disagree

5: somewhat disagree

6: disagree

7: strongly disagree

“False” heading is the number of times a false alert was called

Different tab names indicate the relevant hypothesis from the manuscript that data address.
