# Supplementary material for: Effect of Handler Knowledge of the Detection Task on Canine Search Behavior and Performance
Source: Front Vet Sci. 2020 May 27;7:250. doi: 10.3389/fvets.2020.00250 (PMC7266931; doi:10.3389/fvets.2020.00250)
Supplement: Supplementary file 3 [file Data_Sheet_1.docx]

Supplementary Material

Survey

Do you get paid to be a dog handler?

- Yes
- No

Are you...

- Law enforcement
- Hobby or sport dog handler (e.g. nosework)
- Military dog handler
- Not law enforcement or military but professional dog handler

Is your dog certified or tested by any organizations outside of yourself?

- Yes
- No

If yes, what is the certifying organization? .

During the last search (the 3rd search), I believed there was going to be a target odor hidden.

- Strongly agree
- Agree
- Somewhat agree
- Neither agree nor disagree
- Somewhat disagree
- Disagree
- Strongly disagree

How frequently does your dog receive training?

- Daily
- 4-6 times a week
- 2-3 times a week
- Once a week
- 2-3 times a month
- once a month
- less than once a month

How frequently does your dog experience training trials or runs with no target odors present?

- Multiple times a training session
- Once a training session
- Every other training session
- Every 3-5 training sessions
- Almost Never
- Never

How long have you been training the dog you participated with?

Enter the number in years to the nearest half year.

________________________________________________________________

How frequently does your dog work or get tested (not routine training)?

- Daily
- Weekly
- Monthly
- A few times a year
- Once a year
- Less than once a year

When you train with your dog, do you know where the odor is hidden before the search?

- Always
- Most of the time
- About half the time
- Sometimes
- Never

When you train your dog, does a judge or other trainer that is within visual contact with yourself or the dog know where the odor is hidden?

- Always
- Most of the time
- About half the time
- Sometimes
- Never

Please describe your dog's alert behavior

________________________________________________________________

What odor (s) is your dog trained to alert to?

________________________________________________________________

What is your age?

- Under 18
- 18 - 24
- 25 - 34
- 35 - 44
- 45 - 54
- 55 - 64
- 65 - 74
- 75 - 84
- 85 or older

Are you

- Male
- Female
- Not specified
- Prefer not to answer

Dog Age (in years to the nearest half year)

________________________________________________________________

Dog gender

- Male
- Female

Overall, my dog's detection work is:

- Extremely good
- Moderately good
- Slightly good
- Neither good nor bad
- Slightly bad
- Moderately bad
- Extremely bad

**For each of the statements below please place a cross in the box that most accurately describes your level of agreement: The answer should reflect the *general personality of the dog*, so for example if a statement applies to your dog in some situations but not others, please make a judgement as to how much you agree**

|  |  | Strongly agree | Mainly agree | Partly agree, partly disagree | Mainly disagree | Strongly disagree | Don’t know / not applicable |
| --- | --- | --- | --- | --- | --- | --- | --- |
| 1 | My dog shows extreme physical signs when excited (e.g. drooling, panting, raising hackles, urination, licking lips, widening of eyes) |  |  |  |  |  |  |
| 2 | When my dog gets very excited it can lead to fixed repetitive behaviour (i.e. an action that is repeated in the same way over and over again), such as tail chasing or spinning around in circles |  |  |  |  |  |  |
| 3 | I would consider my dog to be very impulsive (i.e. has sudden, strong urges to act; acts without forethought; acts without considering effects of actions) |  |  |  |  |  |  |
| 4 | My dog doesn't like to be approached or hugged |  |  |  |  |  |  |
| 5 | My dog becomes aggressive (e.g. growl, snarl, snap, bite) when excited |  |  |  |  |  |  |
| 6 | My dog appears to be 'sorry' after it has done something wrong |  |  |  |  |  |  |
| 7 | My dog does not think before it acts (e.g. would steal food without first looking to see if someone is watching) |  |  |  |  |  |  |
| 8 | My dog can be very persistent (e.g. will continue to do something even if it knows it will get punished or told off) |  |  |  |  |  |  |
| 9 | My dog may become aggressive (e.g. growl, snarl, snap, bite) if frustrated with something |  |  |  |  |  |  |
| 10 | My dog is easy to train |  |  |  |  |  |  |
|  |  | Strongly agree | Mainly agree | Partly agree, partly disagree | Mainly disagree | Strongly disagree | Don’t know / not applicable |
| 11 | My dog is not keen to go into new situations |  |  |  |  |  |  |
| 12 | My dog takes a long time to lose interest in new things |  |  |  |  |  |  |
| 13 | My dog calms down very quickly after being excited |  |  |  |  |  |  |
| 14 | My dog appears to have a lot of control over how it responds |  |  |  |  |  |  |
| 15 | My dog is very interested in new things and new places |  |  |  |  |  |  |
| 16 | My dog reacts very quickly |  |  |  |  |  |  |
| 17 | My dog is not very patient (e.g. gets agitated waiting for its food, or waiting to go out for a walk) |  |  |  |  |  |  |
| 18 | My dog seems to get excited for no reason |  |  |  |  |  |  |

**Positive and negative activation scale for dogs**

For each of the statements below, please place a cross in the box which most accurately describes your level of agreement with how your dog behaves in general in this situation. Please consider whether your dog’s behaviour is of similar intensity and occurs as frequently as described. For example, if in item 2 you dog **always** becomes **a little** excited when it is about to go for a walk, you would mainly agree with the statement.

If your dog has never encountered the situation and you are unable to predict the behaviour, please use the not applicable option

|  | | | | | | | | | Agree strongly | Mainly agree | Partly agree,  partly disagree | Mainly disagree | Disagree strongly | Not applicable |  | Office use only |
| --- | --- | --- | --- | --- | --- | --- | --- | --- | --- | --- | --- | --- | --- | --- | --- | --- |
| 1 | Your dog is rarely frightened | | | | | | | |  |  |  |  |  |  |  |  |
| 2 | Your dog becomes very excited when it is about to go for a walk (e.g. when it sees its lead, or when it hears "walkies", etc.) | | | | | | | |  |  |  |  |  |  |  |  |
| 3 | Your dog movements | is | easily | startled | by | noises | and | / or |  |  |  |  |  |  |  |  |
| 4 | Your dog is very persistent in its efforts to get you to play | | | | | | | |  |  |  |  |  |  |  |  |
| 5 | Your dog shows little interest in its surroundings | | | | | | | |  |  |  |  |  |  |  |  |
| 6 | Your dog appears nervous and / or jumpy for several minutes after it has been startled | | | | | | | |  |  |  |  |  |  |  |  |
| 7 | Your dog is easily excited | | | | | | | |  |  |  |  |  |  |  |  |
| 8 | Your dog has a specific fear or phobia | | | | | | | |  |  |  |  |  |  |  |  |
| 9 | Your dog tries to escape from the garden | | | | | | | |  |  |  |  |  |  |  |  |
| 10 | Your dog appears calm in noisy, crowded places | | | | | | | |  |  |  |  |  |  |  |  |
| 11 | Your dog is full of energy | | | | | | | |  |  |  |  |  |  |  |  |
| 12 | Your dog is frightened by noises from the television or radio | | | | | | | |  |  |  |  |  |  |  |  |

|  | | Agree strongly | Mainly agree | Partly agree, partly disagree | Mainly disagree | Disagree strongly | Not applicable |  | Office use only |
| --- | --- | --- | --- | --- | --- | --- | --- | --- | --- |
| 13 | Your dog usually appears relaxed |  |  |  |  |  |  |  |  |
| 14 | Your dog is lazy |  |  |  |  |  |  |  |  |
| 15 | Your dog adapts quickly to changes in its environment (eg. being cared for by different people, moving house or a family member leaving home) |  |  |  |  |  |  |  |  |
| 16 | Your dog appears afraid of the vacuum cleaner or any other familiar household appliance |  |  |  |  |  |  |  |  |
| 17 | Your dog requires a great deal of encouragement to take part in energetic activities |  |  |  |  |  |  |  |  |
| 18 | Your dog persists in being naughty despite being told off for the behaviour |  |  |  |  |  |  |  |  |
| 19 | Your dog appears calm in unfamiliar environments |  |  |  |  |  |  |  |  |
| 20 | Your dog is very boisterous |  |  |  |  |  |  |  |  |
| 21 | Your dog appears unsettled by changes to its routine (e.g. if it is not fed at the usual time, if it is left alone for longer than usual) |  |  |  |  |  |  |  |  |
